# PSPICE Hybrid Modeling and Simulation of Capacitive Micro-Gyroscopes

**DOI:** 10.3390/s18041006

**Published:** 2018-03-28

**Authors:** Yan Su, Xin Tong, Nan Liu, Guowei Han, Chaowei Si, Jin Ning, Zhaofeng Li, Fuhua Yang

**Affiliations:** 1 State Key Laboratory of Superlattices and Microstructures, Institute of Semiconductors, Chinese Academy of Sciences, Beijing 100083, China; suyan16@semi.ac.cn (Y.S.); tongxin16@semi.ac.cn (X.T.); 2 College of Materials Science and Opto-Electronic Technology, University of Chinese Academy of Sciences, Beijing 100049, China; liunan16@semi.ac.cn; 3 Engineering Research Center for Semiconductor Integrated Technology, Institute of Semiconductors, Chinese Academy of Sciences, Beijing 100083, China; hangw1984@semi.ac.cn (G.H.); ningjin@semi.ac.cn (J.N.); 4 State Key Laboratory of Transducer Technology, Chinese Academy of Sciences, Beijing 100083, China; 5 School of Electronic, Electrical and Communication Engineering, University of Chinese Academy of Sciences, Beijing 100049, China; 6 School of Microelectronics, University of Chinese Academy of Sciences, Beijing 100049, China

**Keywords:** prototype development, PSPICE, hybrid model, simulation, MEMS gyroscopes, temperature compensation, optimization, closed-loop

## Abstract

With an aim to reduce the cost of prototype development, this paper establishes a PSPICE hybrid model for the simulation of capacitive microelectromechanical systems (MEMS) gyroscopes. This is achieved by modeling gyroscopes in different modules, then connecting them in accordance with the corresponding principle diagram. Systematic simulations of this model are implemented along with a consideration of details of MEMS gyroscopes, including a capacitance model without approximation, mechanical thermal noise, and the effect of ambient temperature. The temperature compensation scheme and optimization of interface circuits are achieved based on the hybrid closed-loop simulation of MEMS gyroscopes. The simulation results show that the final output voltage is proportional to the angular rate input, which verifies the validity of this model.

## 1. Introduction

In recent years, due to a rapid development of microelectromechanical systems (MEMS) technology, MEMS gyroscopes have become an indispensable portion of the inertial navigation, military, and consumer electronics market for the advantages of their small size, light weight, low cost, and high reliability [1]. The MEMS gyroscope is a device for angular rate detection, and its basic principle is based on the Coriolis Effect [1]. The proof mass of a gyroscope oscillates along the drive axis with a stable frequency and stationary amplitude; meanwhile, this gyroscope is subjected to an angular rate *Ω* in the input axis. Then, it will generate a vibration of the same frequency along the sense axis due to the Coriolis force. The angular rate information is provided by the sense axis, which specifically is proportional to the direct current (DC) output of the sense axis. It is necessary to note that the three axes mentioned above are orthogonal to each other.

MEMS gyroscopes have achieved significant commercial success and have benefited from better and cheaper manufacturing technology. Although the final cost of gyroscopes from mass commercial production is relatively low, it should be noted that prototype design and manufacture and repeat test experiments are costly and consume time. Therefore, building an accurate and complete model is of great significance for reducing the cost of prototype development and guiding the design of peripheral interface electronics. The most commonly used model is the dynamic model, and a good deal of literature [2,3,4,5,6] reveals that the drive mode and sense mode of MEMS gyroscopes can be considered as a second-order Spring-Mass-Damping system. The dynamic model converts the driving force to displacement by the transfer function. The advantage of this model is its intuitive and convenient representation of typical mechanical parameters of MEMS gyroscopes, such as the elastic coefficient of cantilevers and the damping coefficient of the system. Nevertheless, only the displacements of gyroscopes are considered and used to make a system-level simulation, which ignores the actual capacitance changes and the connection to subsequent interface electronics. Considering that micro-Gyroscopes also have characteristic parameters, such as resonant frequency and the quality factor of general resonators, the pure circuit model is also commonly used. In [7], a gyroscope is simplified to a series Resistance-Inductance-Capacitance (RLC) circuit model, where the resonant frequency and quality factor are determined by the size of resistance, inductance, and capacitance in the circuit. This model is easy to implement and it is convenient to combine the RLC model with subsequent interface electronics. However, this model cannot demonstrate some of the mechanical characteristics of MEMS gyroscopes, such as the elastic coupling force and quadrature error. Another novel model is the MIMO T-S fuzzy model, which is built based on nonlinear characteristics of MEMS gyroscopes [8]. The model can strengthen the robustness of the control system with an adaptive sliding controller when external disturbances exist. Nonetheless, this model is non-intuitive and it is challenging to implement the connection between it and subsequent interface electronics.

In this paper, a hybrid model for MEMS gyroscopes is firstly established based on a PSPICE Simulation Model, including two crucial aspects for modeling a micro-gyroscope device, namely different modules in gyroscopes and a principle diagram for them. Then, systematic simulations of the model itself are conducted. At last, hybrid simulations of the model and interface circuits are implemented to achieve closed-loop control of MEMS gyroscopes and optimization of the circuits. The contribution of the present work lies in three aspects.

(1) A detailed model of MEMS gyroscopes is developed. This model considers mechanical thermal noise equivalent disturbance as well as the capacitance model without approximation. The model also considers the effect of temperature on the displacements of both modes.
(2) All output ports of the model are capacitive interfaces, which can be directly connected to conditioning circuits. Therefore, a closed-loop simulation of the model and interface circuits is achieved. A calibration scheme for temperature is developed based on a different Zero Rate Output (ZRO) at different temperatures.
(3) Based on the simulation results, optimization designs of interface circuits are achieved, including the value of the demodulation signal phase $\varphi_{y}$ and the circuit gain *k* in the closed-loop detection circuit.

The remainder of this paper is organized as follows. In Section 2, PSPICE models of different modules in gyroscopes are firstly proposed, including a sensitive structure model, a Coriolis force and elastic coupling force model, a mechanical thermal noise equivalent disturbance model, a mechanical model, the temperature models of gyroscope parameters, and a differential capacitance model. Then, the PSPICE device model of capacitive MEMS gyroscopes is established based on these modules together with its principle diagram. Lastly, the interface circuits of the closed-loop simulation are also discussed. In Section 3, a systematic simulation of this model and a closed-loop simulation of MEMS gyroscopes are conducted with an analysis of the effect of ambient temperature and optimization of interface circuits, and simulation results verify the validity of this model. In Section 4, the corresponding discussion is given.

## 2. Methods

### 2.1. PSPICE Models of Different Modules in MEMS Gyroscopes

**Figure 1:**
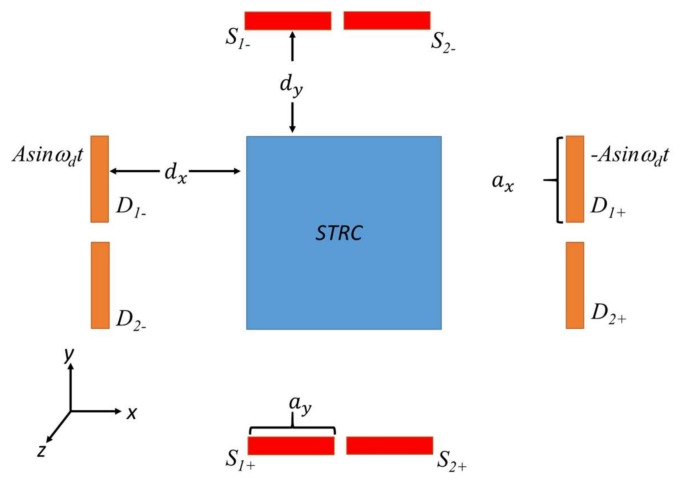
The capacitance interface of the modeled gyroscope.

A complete MEMS gyroscope is a system of coupled mechanical and electrical components. Its capacitance interface is used to achieve the electromechanical coupling process. Figure 1 shows the capacitance interface of the modeled gyroscope.

In Figure 1, the *x*-axis is the direction of drive mode. The *y*-axis is the direction of sense mode, and the *z*-axis is the input direction of the angular rate. The blue movable electrode is the structure (*STRC*) electrode, and it is biased with a DC voltage *U*_0_. In terms of drive mode, the four orange plates are exactly the same and fixed. The upper two plates are actuation electrodes *D*_1+_ and *D*_1−_. *Asinω_d_t* and −*Asinω_d_t* are differential alternating current (AC) excitation voltages applied to the two electrodes for generating an electrostatic driving force *F_x_* on the movable plate. The lower two plates *D*_2+_ and *D*_2−_ are detection electrodes of drive mode, which detect the drive-axis displacement of the *STRC* electrode. The same structure exists in sense mode. The plates *S*_1−_ and *S*_1+_ are detection electrodes of the sense mode, which detect the sense-axis displacement of the *STRC* electrode. The plates *S*_2−_ and *S*_2+_ are force feedback electrodes of sense mode and are used to compensate for the Coriolis force induced by the input angular rate.

**Figure 2:**
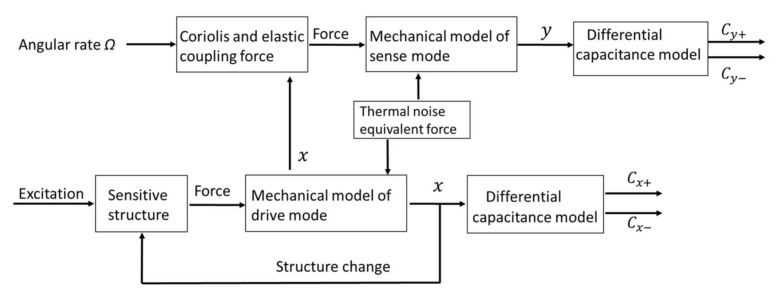
The principle diagram of capacitive microelectromechanical systems (MEMS) gyroscopes.

There is some software that can be used in the simulation of MEMS gyroscopes, such as COMSOL, SIMULINK, and PSPICE. COMSOL mainly emulates the mechanical characteristics of MEMS gyroscopes by finite element analysis, so it is incapable of simulating a complete gyroscope system including the interface circuits. Large quantities of simulations on MEMS gyroscopes are conducted in SIMULINK. In [9], a behavioral model of MEMS gyroscopes constructed in SIMULINK is presented. This model simply describes the mechanical model of gyroscopes and behavioral models of interface circuits. However, SIMULINK software is inadequate to simulate actual circuit electronics and changes of environmental parameters, such as ambient temperature. Because of its good convergence, PSPICE software is suitable for system-level and circuit-level simulation. PSPICE has rapid and accurate simulation ability, so it has been successfully used in a wide variety of linear and nonlinear electrical circuit simulations [10]. PSPICE can simulate both the electronic and non-electronic components, which is consistent with the purpose of this paper, namely the modeling of MEMS gyroscopes and the simulation of interface circuits. Other important reasons to choose PSPICE as the simulation tool are that PSPICE uses more accurate and realistic analog electronic component models and the parameters of models are variable in the simulation process. Figure 2 shows the principle diagram of capacitive MEMS gyroscopes, which is based on the diagram of MEMS sensors in [11]. Different modules of MEMS gyroscopes in this principle diagram are established by a PSPICE Simulation Model, and these modules are discussed separately in the following sections. This diagram illustrates that the inputs of gyroscopes include the AC excitation voltage for drive mode, the input angular rate for sense mode, and the mechanical thermal noise existing in both modes. The outputs of gyroscopes are the changes of differential capacitance, which are caused by the vibration displacements of both modes.

#### 2.1.1. The Sensitive Structure

For drive mode, the electrostatic driving force is induced by the differential AC excitation voltages acting on the sensitive structure. Specifically, *Asinω_d_t* and −*Asinω_d_t* are applied to the actuation electrodes *D*_1+_ and *D*_1−_. Additionally, the *STRC* electrode is biased with a DC voltage *U*_0_. This electrostatic force *F_x_* is normal and it is expressed as a gradient of the electrical potential energy in the capacitor.

The energy of a parallel plate capacitor is

(1) $$E=\frac{1}{2}CV^{2},$$

where *C* is the capacitance value and *V* is the voltage applied to the capacitor. The electrostatic driving force *F_x_*_+_ between the movable plate and the right actuation plate can be calculated as follows:

(2) $$F_{x+}=\frac{\partial E}{\partial x}=\frac{C_{0}V^{2}}{2d_{x}}=\frac{1+\frac{x}{d_{x}}}{1-\frac{x}{d_{x}}},$$

where *d_x_* is the initial spacing of the two plates, *x* is the drive-axis displacement of the *STRC* electrode, *C*_0_ is initial capacitance between the movable plate and actuation plates of drive mode and its value is $\frac{\varepsilon a_{x}}{d_{x}}$, *ε* is the dielectric constant, and *a_x_* is the plate area.

The same derivation is also applied to the left actuation plate and the movable plate. Additonally, the total electrostatic driving force *F_x_* for the movable plate is shown below:

(3) $$F_{x}=\frac{{2C}_{0}}{d_{x}}\;\frac{1}{\left(1+\frac{x}{d_{x}})(1-\frac{x}{d_{x}}\right)}\;\left({\mathrm{Asin}\omega }_{d}t+\frac{U_{0}x}{d_{x}}\right)\left(U_{0}+\frac{x}{d_{x}}{\mathrm{Asin}\omega }_{d}t\right).$$

The above formula shows that *F_x_* is related to *Asinω_d_t* and *x*. In case of *x* << *d*, *F_x_* can be simplified as follows:

(4) $$F_{x}=\frac{{2C}_{0}}{d_{x}}{\mathrm{AU}}_{0}{\sin\omega }_{d}t.$$

Formula (4) indicates that *F_x_* is proportional to the excitation voltage *Asinω_d_t* when *x* << $d_{x}$. Additionally, the gain factor *k_vf_* is $\frac{{2C}_{0}}{d_{x}}U_{0}$. With the sensitive structure, the AC excitation voltage can be converted into the electrostatic driving force of drive mode. The scale factor *k_vf_* will be used in the closed-loop simulation of MEMS gyroscopes.

#### 2.1.2. The Coriolis Force and Elastic Coupling Force Model

This model describes the mechanism for the generation of Coriolis force and elastic coupling force. The Coriolis force is induced by the Coriolis Effect. It is related to the angular rate input and the displacement velocity of drive mode. The Coriolis force can be calculated according to the specific formula,

(5) $${\vec{F}}_{\mathrm{Coriolis}}=-{2m}_{x}(\vec{\Omega }\times {\vec{V}}_{x}),$$

where $\vec{\Omega }$ is the angular rate of rotation and is along the *z*-axis, *V_x_* is the displacement velocity of the *STRC* electrode in drive mode, and *m_x_* is the effective mass of drive mode, which also happens to be the proof mass in our gyroscope model. The direction of the Coriolis force is along the *y*-axis. It should be noted that when the input angular rate is relatively large, the Coriolis force generated by the movement of sense mode will also affect the drive mode. The corresponding Coriolis force is

(6) $${\vec{F}}_{\mathrm{Coriolis}}=-2m_{x}(\vec{\Omega }\times {\vec{V}}_{y}),$$

where *V_y_* is the displacement velocity of the *STRC* electrode in sense mode. This force will act upon the drive mode of MEMS gyroscopes.

MEMS gyroscopes may be affected by fabrication imperfections which can lead to a coupling error between the drive mode and the sense mode. The most common error is a quadrature error due to spring imbalance, which causes an additional elastic coupling force. This force is orthogonal to the Coriolis force in the phase [12], and it is proportional to coupling stiffness and drive-axis displacement. The size of a coupling force can be calculated as

(7) $$F_{\mathrm{coupling}}=k_{\mathrm{yx}}x,$$

where *k_yx_* is the coupling stiffness, which is used to characterize the effect of spring imbalance. The direction of an elastic coupling force is also along the *y*-axis.

#### 2.1.3. The Mechanical Thermal Noise Equivalent Disturbance

For the mechanical thermal noise existing in a MEMS gyroscope’s structure, [13] indicates that the effect of the noise can be equivalent to a constant disturbance force upon both modes. The size of the equivalent force is

(8) $$F_{n}=\sqrt{{4K}_{B}T_{0}\mathrm{cB}},$$

where $K_{B}$ is Boltzmann’s constant, and its value is 1.38 × 10^−23^ J/K, $T_{0}$ is degrees Kelvin, and *B* is the bandwidth of the gyroscope. For a mode-split gyroscope, its bandwidth is approximately equal to 0.54 times the frequency difference when operating in open-loop detection mode [14]. Additionally, this bandwidth is valid for the simulation of a PSPICE device model of capacitive MEMS gyroscopes. *c* is the damping coefficient of the gyroscope structure, and it is different for the two modes. The corresponding values of the damping coefficient are shown below:

(9) $$c_{\mathrm{xx}}=\frac{m_{x}\omega_{x}}{Q_{x}},$$

(10) $$c_{\mathrm{yy}}=\frac{m_{y}\omega_{y}}{Q_{y}},$$

where *Q_x_* and *ω_x_* are the quality factor and resonant angle frequency of drive mode, respectively, *Q_y_* and *ω_y_* are the quality factor and resonant angle frequency of sense mode, respectively, and *m_x_* and *m_y_* are the effective drive mass and effective sense mass, respectively. It is noteworthy that the quality factor and resonant frequency of gyroscopes are greatly affected by ambient temperature. The effect of temperature on parameters will be discussed in the following temperature model.

#### 2.1.4. The Mechanical Model of MEMS Gyroscopes

A mechanical model of MEMS gyroscopes can convert the resultant forces into displacements for both modes. Additionally, a gyroscope can be considered as a second-order Spring-Mass-Damping system. Its characteristics equations are

(11) $$m_{x}\ddot{x}+c_{\mathrm{xx}}\dot{x}+k_{\mathrm{xx}}x+F_{\mathrm{nx}}=F_{x}+2m_{x}\Omega \dot{y},$$

(12) $$m_{y}\ddot{y}+c_{\mathrm{yy}}\dot{y}+k_{\mathrm{yy}}y+F_{\mathrm{ny}}=-{2m}_{x}\Omega \dot{x},$$

where *k_xx_* and *k_yy_* are the stiffness coefficients of the drive mode and sense mode, respectively, *F_nx_* and *F_ny_* are the mechanical thermal noise equivalent disturbance force of the drive mode and sense mode, respectively, and *F_x_* and $-{2m}_{x}\Omega \dot{x}$ are the driving forces of the two modes, respectively. The transfer functions of the characteristic equations are

(13) $$H_{x}\left(s\right)=\frac{1}{m_{x}s^{2}+d_{\mathrm{xx}}s+k_{\mathrm{xx}}}=\frac{1}{m_{x}s^{2}+\frac{\omega_{x}m_{x}}{Q_{x}}s+m_{x}{\omega_{x}}^{2}},$$

(14) $$H_{y}\left(s\right)=\frac{1}{m_{y}s^{2}+d_{\mathrm{yy}}s+k_{\mathrm{yy}}}=\frac{1}{m_{y}s^{2}+\frac{\omega_{y}m_{y}}{Q_{y}}s+m_{y}{\omega_{y}}^{2}},$$

where *s* is the variable symbol *jω_d_* of the transfer function. The transfer functions convert the corresponding resultant forces into displacements for both modes when mechanical model parameters of gyroscopes are known. Additionally, the mechanical model is also related to temperature.

#### 2.1.5. The Temperature Models of Gyroscope Parameters

As discussed earlier, the quality factor and resonant frequency of gyroscopes are greatly impacted by ambient temperature. Therefore, the temperature will affect the displacements of both modes. The authors in [15] indicate that temperature and resonant frequency approximate a linear relationship. A corresponding experiment was carried out to obtain the linear factor based on the gyroscopes in the authors’ research group. The varying resonant frequency measurement is based on the dynamic signal analyzer. At different temperatures, the phase-locked loop controls the drive mode to operate at different working frequencies. Because the *Q* value of drive mode is very large, the working frequency is approximately equal to the resonant frequency. Therefore, the resonant frequencies of drive mode are obtained by measuring the working frequencies through the “channel” port of the dynamic signal analyzer at different ambient temperatures. For sense mode, the measurement of resonant frequency at different temperatures is achieved through the “source” and “channel” ports of the dynamic signal analyzer. The “source” port outputs a chirp sweep signal to the force feedback electrode. Additionally, there are corresponding response signals with different amplitudes on the detection electrode. These response signals are displayed on the dynamic signal analyzer through the “channel” port. The point with the largest response amplitude corresponds to the resonant frequency of sense mode. Then, the relationship between the resonant frequency and the ambient temperature is shown as follows

(15) $$f_{x}=-0.1274\left(T-27\right)+10.157k,$$

(16) $$f_{y}=-0.1287\left(T-27\right)+10.257k,$$

where $f_{x}$ and $f_{y}$ are the resonant frequency of drive mode and sense mode, respectively, and *T* is degrees centigrade.

In order to obtain a higher quality factor, the microstructure of MEMS gyroscopes is vacuum-packaged. In [16], the quality factor is proportional to the resonant frequency and inversely proportional to the square root of the degrees kelvin. Additionally, the proportional factors of both modes are different. The resonant frequency and quality factor at 27 degrees centigrade will be used to calculate the proportional factor of both modes. Therefore, the *Q* values of two modes at 27 degrees Celsius need to be measured. The *Q* value measurement needs the “source” port and the “channel” port of the dynamic signal analyzer. For the drive mode and sense mode, the “source” port outputs a chirp sweep signal to the actuation electrode and the force feedback electrode, respectively. The corresponding response signals are generated on the detection electrodes of both modes and they are displayed on the dynamic signal analyzer through the “channel” port. The *Q* value is obtained through dividing the resonant frequency by the corresponding −3 dB bandwidth. A corresponding experiment was carried out to obtain the linear factor based on the gyroscopes in the authors’ research group and the formulas are shown as follows:

(17) $$Q_{x}=255.856f_{x}/\sqrt{\left(T+273.15\right)},$$

(18) $$Q_{y}=16.891f_{y}/\sqrt{\left(T+273.15\right)}.$$

#### 2.1.6. The Differential Capacitance of MEMS Gyroscopes

In Figure 1, the lower two orange plates and the movable blue electrode form the differential detection capacitance of drive mode, and the capacitance is, respectively:

(19) $$C_{x+}=\frac{{\varepsilon a}_{x}}{d_{x}-x},$$

(20) $$C_{x-}=\frac{{\varepsilon a}_{x}}{d_{x}+x}.$$

For sense mode, its differential detection capacitance is formed between the two fixed red plates and the movable blue electrode. The differential capacitance is, respectively:

(21) $$C_{y+}=\frac{{\varepsilon a}_{y}}{d_{y}-y},$$

(22) $$C_{y-}=\frac{{\varepsilon a}_{y}}{d_{y}+y},$$

where *d_y_* is the initial spacing of the capacitance, *a_y_* is plate area, and *y* is the sense-axis displacement of the *STRC* electrode. In our capacitance model, the actual parallel-plate detection mechanism is used. The capacitance change is not simply approximated to a direct proportion of the displacement. This approximation is sometimes used in the simulation of MEMS sensors. The distance of the MEMS capacitor plate is much less than its length and width. Therefore, the edge effect of capacitance can be ignored. The parallel-plate sensing mechanism contributes a nonlinear behavior between the sense capacitance and the sense-axis displacement. This nonlinearity will be eliminated in normal operation because the displacement produced by the Coriolis force is suppressed by the feedback force. In actual gyroscopes, the capacitive nonlinearity is from multiple effects, including microfabrication process errors, parallel plate nonlinearity due to deformation, C-V conversion circuit behavior, and the quadrature error existing in gyroscopes. In our model, these factors are assumed to be ideal because the entire simulation is based on the PSPICE’s own model components and electronics.

### 2.2. Establishment of Capacitive MEMS Gyroscopes PSPICE Device Model

The parameters of the PSPICE device model of parallel-plate capacitive micro-gyroscopes are shown in Table 1 at a constant temperature of 27 degrees centigrade. All other parameters except $k_{\mathrm{yx}}$ are derived from the gyroscopes in the authors’ research group. The data for $k_{\mathrm{yx}}$ in Table 1 is not experimental data but simulation data. The authors in [4,17] indicate that the typical quadrature force is much larger than the Coriolis force used for sensing the external angular rate. In the case of other parameters, they are known assuming that $k_{\mathrm{yx}}$ = 5 N/m is in accordance with the precondition (assuming the typical angular rate *Ω* is 0.01°/s). Additionally, $k_{\mathrm{yx}}$ = 5 N/m is experimental data according to [4]. Therefore, we take $k_{\mathrm{yx}}$ = 5 N/m as our simulation data.

**Figure 3:**
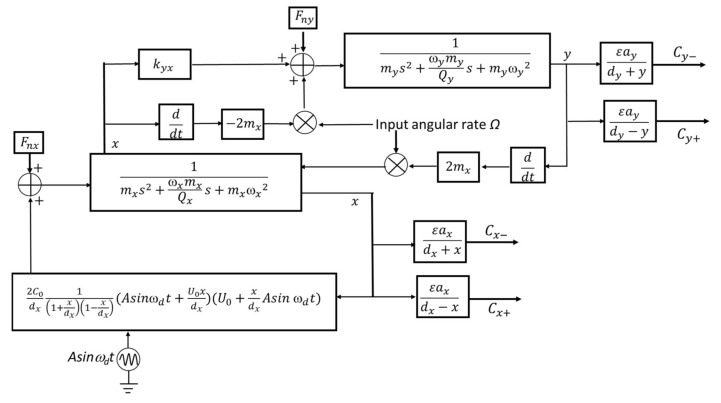
The PSPICE device model of capacitive micro-gyroscopes.

The PSPICE device model is established according to Figure 2 as shown in Figure 3. The formulas in Figure 3 represent the modules in Figure 2 and they have been discussed in detail in Section 2.1.

This model consists of drive mode and sense mode. In terms of drive mode, the resultant force and mechanical model form a loop to generate a displacement. All of the output ports are differential capacitance interfaces that can be directly connected to a C-V conversion circuit. For the sense mode, the driving force is induced by the Coriolis force and elastic coupling force model. It will be converted into a displacement with the mechanical model. The displacement affects the differential capacitance. The final differential capacitance output is related to the angular rate input. The specific simulation results of a parallel-plate capacitive micro-gyroscopes PSPICE model at a constant temperature of 27 degrees centigrade are shown in Section 3.

The temperature will affect the resonant frequency and quality factor, which changes the gyroscope’s mechanical model. So, the gyroscope will have different motion states at different temperatures. The simulation results at different temperatures with the same excitation voltage are also described in Section 3.

### 2.3. The PSPICE Closed-Loop Simulation of MEMS Gyroscopes

**Figure 4:**
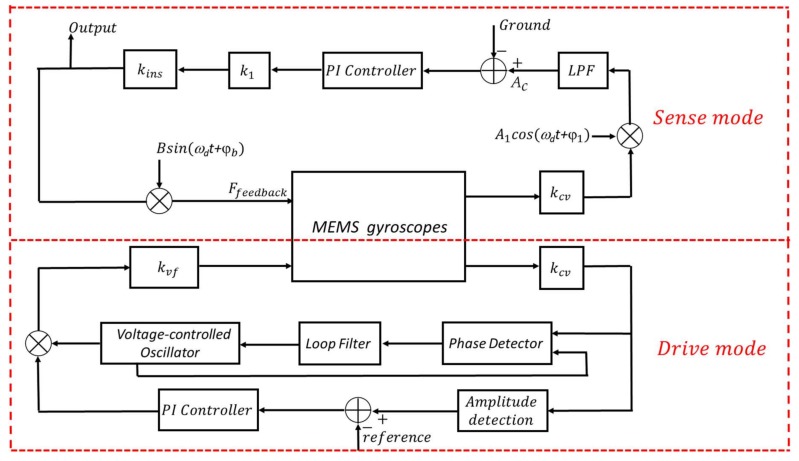
The closed-loop simulation diagram of MEMS gyroscopes.

The temperature will change the motion state of a gyroscope, so it is necessary to implement the closed-loop control of MEMS gyroscopes. For drive mode, the Phase-locked Loop (PLL) method [18] is usually used to control the drive frequency’s stability. The Automatic Gain Control (AGC) method [19] is used to keep the amplitude of the drive-axis displacement constant. For sense mode, the Closed-loop Detection method [20] is used to ensure that the proof mass always vibrates near the equilibrium position. The corresponding block diagram of the closed-loop simulation based on the above three methods and the micro-gyroscopes PSPICE model is shown in Figure 4.

For drive mode, the control circuits consist of a C-V conversion circuit, a PLL frequency control circuit, and an AGC amplitude control circuit. The gain factors *k_vf_* and *k_cv_* represent the sensitive structure and the C-V conversion circuit, respectively. When the drive mode is stable, the output of the loop filter should be a stabilized value at a fixed temperature, which means that the vibration frequency of displacement reaches stability. It is noteworthy that the stabilized value will change slightly with the ambient temperature due to the change of resonant frequency. Additionally, the output of the amplitude detection module is equal to the reference value when the drive mode is stable, which means that the vibration amplitude of the displacement reaches a constant value. The corresponding simulation results of closed-loop control are shown in Section 3.

For sense mode, the purpose of closed-loop detection is to suppress the Coriolis force completely. This is accomplished by adding differential AC signals to the feedback electrodes of sense mode, namely $S_{2+}$ and $S_{2-}$ in Figure 1. It is noteworthy that the phase of the differential AC signal needs to be opposite to the phase of the displacement velocity of drive mode. A corresponding feedback force is induced to suppress the Coriolis force by adjusting the amplitude of the differential AC signals. The feedback force can be calculated by formula (4), and its value is

(23) $$F_{\mathrm{feedback}}=\frac{{2C}_{1}}{d_{y}}U_{0}\mathrm{Bsin}\left(\omega_{d}t+\varphi_{b}\right)=k_{\mathrm{ins}}\mathrm{Bsin}\left(\omega_{d}t+\varphi_{b}\right),$$

where *C*_1_ is initial capacitance between the movable *STRC* and each of the four fixed plates of the sense mode, $\mathrm{Bsin}\left(\omega_{d}t+\varphi_{b}\right)$ is the AC signal, which has opposite phase of the displacement velocity of drive mode, and $k_{\mathrm{ins}}$ is the simplified interface gain of the force feedback structure. In our model, the output of the $k_{\mathrm{ins}}$ gain module is the output voltage of MEMS gyroscopes corresponding to different angular rates.

## 3. Results

### 3.1. The Simulation Results of the Capacitive MEMS Gyroscopes PSPICE Device Model

**Figure 5:**
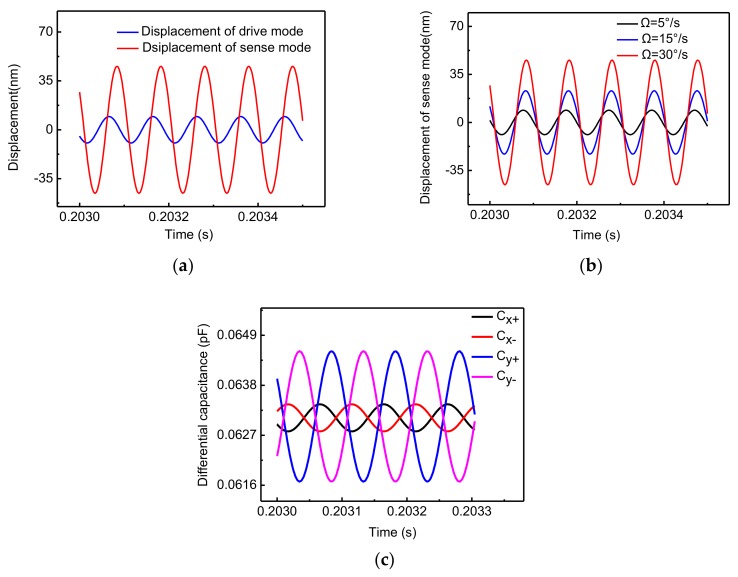
The simulation results of the capacitive micro-gyroscopes PSPICE model: (**a**) drive-axis displacement and sense-axis displacement, *Ω* = 30°/s (**b**) sense-axis displacements at different angular rates (**c**) the differential detection capacitance of drive mode and sense mode, *Ω* = 30°/s.

The simulation results of the capacitive micro-gyroscopes PSPICE model are described in Figure 5. Figure 5a shows that the drive-axis displacement and sense-axis displacement are stable sine waves with the same frequency as the AC excitation voltage. This means that the Coriolis Effect has been achieved through the established PSPICE model. Figure 5b indicates that the amplitude of the sense-axis displacement changes with the angular rate. It is noteworthy that the sense-axis displacements corresponding to different angular rates will intersect at specific points. At these points, the phases of the sense-axis displacement are some periodic values, which will cause the sense-axis displacement to be zero and the quadrature displacement to be the maximum. Therefore, these points represent the quadrature displacement, which is induced by an elastic coupling force and its amplitude is 4.804 nm. The sense-axis displacement is not proportional to the angular rate input due to the existence of quadrature displacement. Figure 5c shows that the displacements of gyroscopes can be converted into differential capacitance changes. Thus, all output ports of this PSPICE model are capacitance interfaces that can be connected directly to a C-V conversion circuit.

**Figure 6:**
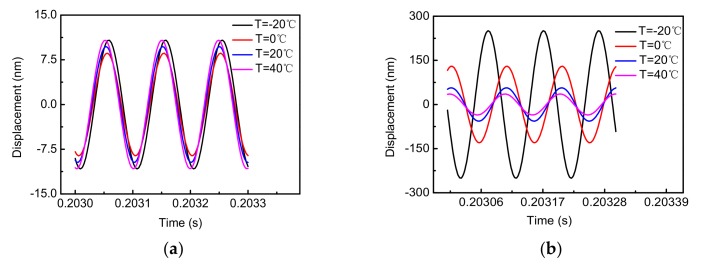
Displacements of the capacitive MEMS gyroscopes PSPICE model at different temperatures with the same excitation ($f_{d}=10.157\;\mathrm{kHz},\;A=5\;V$): (**a**) drive-axis displacement (**b**) sense-axis displacement.

Changes in ambient temperature will cause changes in the resonant frequencies and quality factors of MEMS gyroscopes, which can affect the dynamic characteristics of the gyroscope. Therefore, the same excitation voltage will induce different drive-axis displacement and sense-axis displacement at different ambient temperatures. Figure 6 shows the corresponding simulation results.

The simulation results show that the drive-axis displacement and sense-axis displacement will have different phases and amplitudes at different temperatures, which indicates that the gyroscope is greatly affected by the ambient temperature. Therefore, subsequent conditioning circuits are necessary to achieve the closed-loop control of MEMS gyroscopes at different ambient temperatures.

### 3.2. The Simulation Results of the Closed-Loop Control of MEMS Gyroscopes

#### 3.2.1. The Closed-Loop Simulation Results of Drive Mode

**Figure 7:**
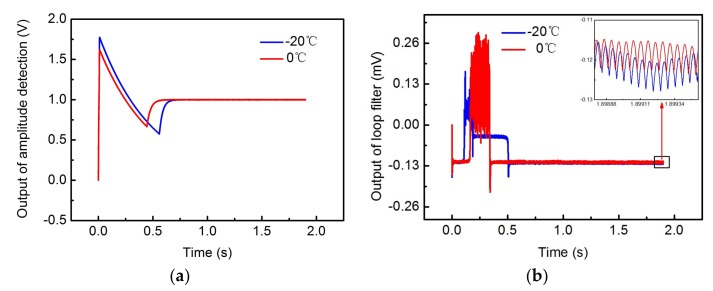
The closed-loop simulation results of drive mode: (**a**) the output voltage of amplitude detection at different temperatures (**b**) the output voltage of the loop filter at different temperatures.

Figure 7 shows the simulation results of drive mode at different temperatures.

The simulation results show that the outputs of amplitude detection at different temperatures are all 1 V, which is equal to the reference value. This means that the amplitude of the drive-axis displacement is a fixed value as the excitation voltage is adjusted at different ambient temperatures. Additionally, at different temperatures, the output of the loop filter reaches a stabilized value at about 0.5 s. This means that the vibration frequency of the drive-axis displacement has stabilized. Therefore, the drive mode realizes closed-loop control of the vibration amplitude and frequency at different temperatures. It is noteworthy that the output voltages of the loop filter corresponding to different temperatures are different, which indicates that the stable vibration frequency of the drive-axis displacement is different when the ambient temperature changes. This is because the resonant frequency of drive mode is affected by the ambient temperature.

#### 3.2.2. The Closed-Loop Simulation Results of Sense Mode

**Figure 8:**
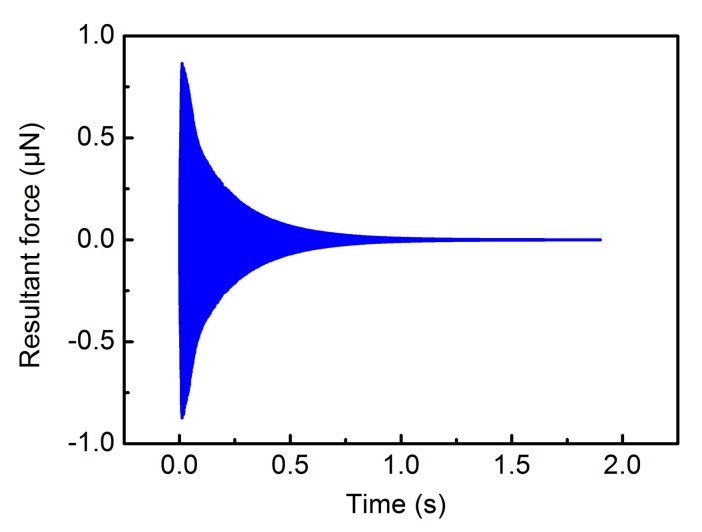
The resultant force of Coriolis force and feedback force.

For sense mode, the closed-loop detection is based on complete compensation for the Coriolis force. This means that the proof mass is only affected by the quadrature force, which causes the proof mass to always vibrate near the equilibrium position. The closed-loop detection method uses the Proportion-Integration (PI) controller algorithm, which will be specifically explained in Section 3.2.3. Figure 8 shows the simulation result of the resultant force of Coriolis force and feedback force.

**Figure 9:**
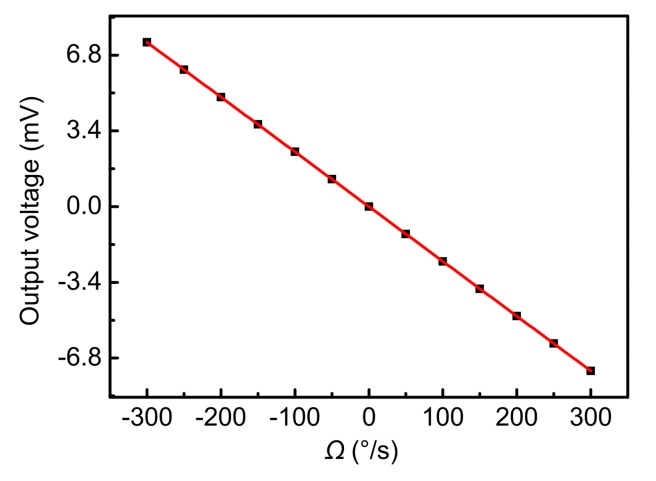
The curve of input angular rate and output voltage.

The simulation result shows that the resultant force is gradually changed to Zero at 1 s (*Ω* = −300°/s), which means that the Coriolis force is completely compensated for by the feedback force. Specifically, the closed-loop detection is achieved by setting up feedback electrodes. The electric potential energy is generated between the feedback electrodes and the proof mass by applying external feedback voltages, so a tangential electrostatic feedback force is generated to compensate for the Coriolis force completely. Therefore, the closed-loop detection is implemented based on this method. Figure 9 shows the output voltage corresponding to different input angular rates.

Different input angular rates can cause different Coriolis force values, so the feedback force induced by the output voltage also changes. Therefore, there is a proportional relationship between the input angular rate and the output voltage. Figure 9 shows that the scale factor is –0.02459 mV/(°/s) with a linear correlation coefficient of 1. The nonlinearity induced by the parallel-plate sensing mechanism is completely eliminated in closed-loop detection because the proof mass always vibrates near the equilibrium position.

**Figure 10:**
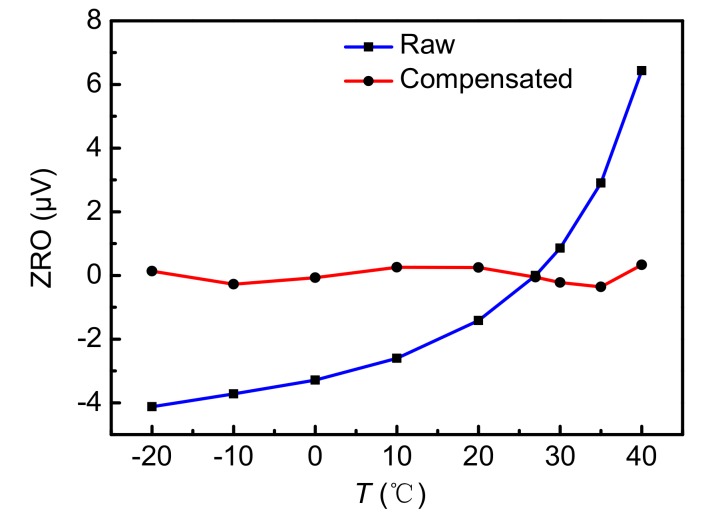
The Zero Rate Output (ZRO) at different temperatures and the temperature compensation curve.

Changes in temperature will cause changes in the motion state of a gyroscope. So, the phase $\varphi_{y}$ of the sense-axis displacement will also be changed. However, the phase $\varphi_{1}$ of the demodulated signal used in the closed-loop detection circuit cannot be changed in real-time. Therefore, different temperatures will induce different output voltages when the input angular rate is zero. Figure 10 shows different ZRO voltages of gyroscopes at different temperatures. Additionally, a corresponding cubic fitting curve based on the self-compensation method [21] is obtained as

(24) $$\mathrm{ZRO}\left(T\right)=0.0001186T^{3}+0.000642T^{2}+0.0175T-3.222,$$

where ZRO(T) is the curve fitting results for *T*, and its unit is μV. The final compensated curve is the difference between the raw simulation result and the fitting result. The final temperature result shows that the compensated ZRO voltages are around zero in the temperature range from −20 to 40 °C.

#### 3.2.3. The Optimization Designs of the Closed-Loop Detection Circuit

**Figure 11:**
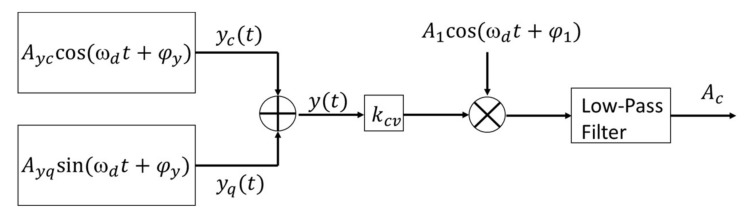
The orthogonal demodulation diagram of closed-loop detection.

It is noteworthy that the choice of the demodulated signal phase $\varphi_{1}$ is important for the aforementioned closed-loop detection scheme. The purpose of closed-loop detection is to control $A_{\mathrm{yc}}=0$ by applying the feedback voltage. This is achieved indirectly by controlling $A_{c}=0$ while also requiring a suitable phase $\varphi_{1}$. $A_{c}$ is the controlled variable of the PI controller. The Coriolis displacement and quadrature displacement can be assumed as

(25) $$y_{\mathrm{Coriolis}}\left(t\right)=A_{\mathrm{yc}}\cos(\omega_{d}t+\varphi_{y}),$$

(26) $$y_{\mathrm{quadrature}}\left(t\right)=A_{\mathrm{yq}}\sin(\omega_{d}t+\varphi_{y}),$$

where $A_{\mathrm{yc}}$ is the amplitude of the Coriolis displacement, and this displacement is the result of the interaction between the Coriolis force and the feedback force, $A_{\mathrm{yq}}$ is the amplitude of the quadrature displacement, and $\varphi_{y}$ is the phase of the sense-axis displacement. The orthogonal demodulation process in the closed-loop detection circuit is shown in Figure 11.

**Figure 12:**
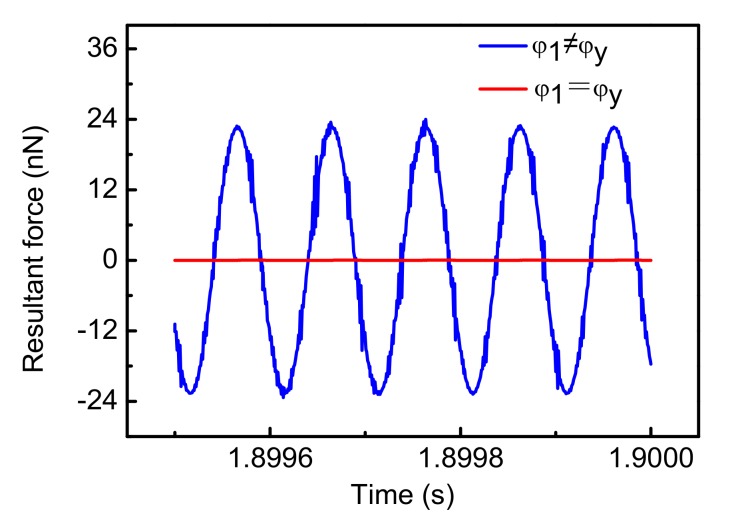
The resultant force at different demodulation phase angles.

Therefore, the controlled variable of the PI controller is

(27) $$A_{c}=\frac{1}{2}k_{\mathrm{cv}}A_{\mathrm{yc}}A_{1}\cos\left(\varphi_{y}-\varphi_{1}\right)+\frac{1}{2}k_{\mathrm{cv}}A_{\mathrm{yq}}A_{1}{\sin(\varphi }_{y}-\varphi_{1}),$$

where *φ*_1_ is the phase of the demodulated signal. When $\varphi_{y}=\varphi_{1}$, the second term of Equation (27) is zero. This means that the quadrature displacement has no effect on the output voltage. Therefore, $A_{c}$ and $A_{\mathrm{yc}}$ are in a ratio relationship. The loop can control that $A_{\mathrm{yc}}$ equals to zero by controlling that $A_{c}$ is equal to zero. When $\varphi_{y}\neq \varphi_{1}$, the error term due to the quadrature displacement is not equal to zero. This means that the output voltage is affected by the Coriolis displacement and quadrature displacement. Therefore, $A_{\mathrm{yc}}$ cannot be controlled to zero by controlling $A_{c}$ to zero. Additionally, the Coriolis force cannot be completely compensated for in this case. In consequence, the choice of the demodulated signal phase is important for the optimization of Coriolis force cancellation. Figure 12 shows the compensation for the Coriolis force at different demodulation phases.

The simulation results show that when $\varphi_{1}\neq \varphi_{y}(\varphi_{1}-\varphi_{y}\approx 130\text{°})$, the resultant force of Coriolis force and feedback force is not equal to zero. Therefore, the Coriolis force has not been completely compensated for in this case. The above analysis and simulation show that the optimization of Coriolis force compensation is achieved by choosing a suitable demodulation signal phase $\varphi_{1}$.

**Figure 13:**
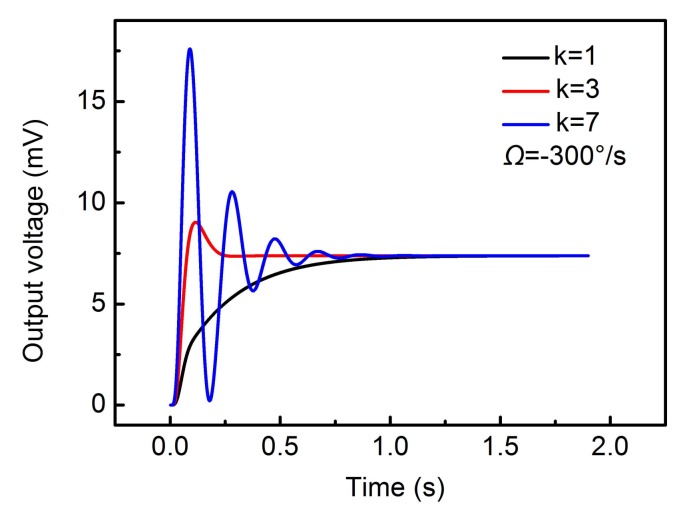
The stabilization time at different circuit gain *k.*

In addition to the phase of the demodulation signal, the circuit gain of closed-loop detection will also affect the performance of the circuit. For simplicity, we take the product of $k_{1}$ and $k_{ins}$ as *k* for analysis. Figure 13 shows different stabilization times at different *k*.

The simulation results show that a suitable circuit gain will induce the shortest stabilization time. The feedback force is proportional to the output voltage. So, the feedback force is constantly changing during the process of stabilization. A large *k* will cause the overshoot of the output voltage to increase, thus increasing the stabilization time of the feedback force. A small *k* will also slow down the response process of the feedback force. So, the corresponding stabilization time will also be increased.

## 4. Discussion

In this paper, a PSPICE device model of parallel-plate capacitive micro-gyroscopes was introduced for reducing the cost of prototype development, which consists of a principle diagram and different modules. Systematic simulations of this model and closed-loop control circuits were conducted, and it is demonstrated that this PSPICE device model has two clear advantages:

(1) Some details of MEMS gyroscopes are considered in this model, including the capacitance model without approximation, mechanical thermal noise, and the effect of temperature.
(2) The closed-loop simulation of MEMS gyroscopes is achieved based on the differential capacitance interfaces of this model and subsequent interface circuits.

Some optimization designs of the circuit have been implemented based on this model, including a complete compensation for Coriolis force and the corresponding stabilization time. The test Printed circuit board (PCB) of closed-loop detection is being designed. The corresponding experiment result will be summarized in future work.

## Figures and Tables

**Table 1:** Micro-gyroscopes PSPICE model simulation parameters (*T =* 27 °C).

Parameter	Value
*f_x_*	10.157 kHz
*f_y_*	10.257 kHz
*f_d_*	10.157 kHz
*U*_0_	6 V
*d_x_* = *d_y_*	2 μm
*ε*	8.85 p
*a_x_* = *a_y_*	14.256 × 10^−9^ m^2^
*m_x_*	12.3 μg
*Q_x_*	150,000
*k_yx_*	5 N/m
*m_y_*	0.245 μg
*Q_y_*	10,000
*A*	5 V
